# Revealing the Chemical Composition of Birch Pollen Grains by Raman Spectroscopic Imaging

**DOI:** 10.3390/ijms23095112

**Published:** 2022-05-04

**Authors:** Clara Stiebing, Nele Post, Claudia Schindler, Bianca Göhrig, Harald Lux, Jürgen Popp, Astrid Heutelbeck, Iwan W. Schie

**Affiliations:** 1 Leibniz Institute of Photonic Technology (Leibniz-IPHT), Albert-Einstein-Straße 9, 07745 Jena, Germany; clara.stiebing@leibniz-ipht.de (C.S.); juergen.popp@leibniz-ipht.de (J.P.); 2 Department of Medical Engineering and Biotechnology, University of Applied Sciences Jena, Carl-Zeiss-Promenade 2, 07745 Jena, Germany; nelte@arcor.de; 3 Institute of Occupational, Social and Environmental Medicine, Jena University Hospital, Erlanger Allee 103, 07747 Jena, Germany; claudia.schindler@med.uni-jena.de (C.S.); bianca.goehrig@med.uni-jena.de (B.G.); h.lux@ruppiner-kliniken.de (H.L.); astrid.heutelbeck@med.uni-jena.de (A.H.); 4 Department of Psychiatry, Psychotherapy and Psychosomatic Medicine, Brandenburg Medical School, 16816 Neuruppin, Germany; 5 Institute of Physical Chemistry and Abbe Center of Photonics, Friedrich Schiller University Jena, Helmholtzweg 4, 07743 Jena, Germany

**Keywords:** vibrational spectroscopy, Betula, allergy, sporopollenin, multiple curve resolution

## Abstract

The investigation of the biochemical composition of pollen grains is of the utmost interest for several environmental aspects, such as their allergenic potential and their changes in growth conditions due to climatic factors. In order to fully understand the composition of pollen grains, not only is an in-depth analysis of their molecular components necessary but also spatial information of, e.g., the thickness of the outer shell, should be recorded. However, there is a lack of studies using molecular imaging methods for a spatially resolved biochemical composition on a single-grain level. In this study, Raman spectroscopy was implemented as an analytical tool to investigate birch pollen by imaging single pollen grains and analyzing their spectral profiles. The imaging modality allowed us to reveal the layered structure of pollen grains based on the biochemical information of the recorded Raman spectra. Seven different birch pollen species collected at two different locations in Germany were investigated and compared. Using chemometric algorithms such as hierarchical cluster analysis and multiple-curve resolution, several components of the grain wall, such as sporopollenin, as well as the inner core presenting high starch concentrations, were identified and quantified. Differences in the concentrations of, e.g., sporopollenin, lipids and proteins in the pollen species at the two different collection sites were found, and are discussed in connection with germination and other growth processes.

## 1. Introduction

Birch pollen is of specific interest because it is one of the most dominant tree pollen types found in Northern and Central Europe, and causes the majority of allergic rhinitis [1]. Numerous environmental factors have been and are being discussed in relationship to the increase in allergic diseases [2,3,4]. According to a publication of the German Federal Environmental Agency [5], experimental and in situ studies show an influence of different environmental factors—such as the atmospheric CO_2_ concentration and air temperature—on pollen and biomass production, respectively, by allergenic plant species. Compared to pre-industrial CO_2_ concentrations, both the current and a predicted 21st century CO_2_ concentration increased pollen and allergen production of *Ambrosia artemisiifolia* L., an invasive plant with high allergenic potential [6,7,8]. Ziska et al. and Song et al. described the higher biomass production of Ambrosia plants at urban compared to rural sites, with urban sites having higher CO_2_ (30 to 31% and 3%, respectively) and air temperature values (1.8 to 2 °C and 1.8 °C, respectively) than rural sites [9,10]. In Europe, the air concentrations of pollen from numerous plant species—some of which are highly allergenic—have increased over the past 30 years, especially in urban areas [11]. To investigate the aforementioned changes, systematic studies implementing analytical methods are needed in order to record biochemical changes in a qualitative and quantitative way. Raman spectroscopy allows the non-destructive identification and characterization of airborne particles such as pollen, and is in this context of special interest, as it also allows molecular imaging on a sub-grain level.

By combining molecular information with subcellular optical resolution, vibrational spectroscopies can fill the gap between destructive modalities, which only provide chemical information from bulk samples, and analytical modalities, e.g., brightfield microscopy, that can give morphological information but lack the chemical specificity. Over the past decade, various studies concerning pollen were performed using Raman and Infrared (IR) spectroscopy [12,13,14,15,16,17]. A small subset of studies dealt with different enhancement techniques, such as surface-enhanced Raman spectroscopy [18,19,20] or resonance-enhanced Raman spectroscopy [21]. The majority of studies focused on the taxonomic classification and their relationships [22,23,24,25,26], and there were few studies on the differentiation due to, e.g., growth and environmental conditions [27,28,29] or nutritional parameters [30]. Recently, efforts towards a rapid and automatic identification of pollen were made based on the reduction of the spectral dataset into specific band regions [31], or on the utilization of a high-throughput IR system [32]. An automated high-throughput Raman system was specifically developed to fulfill major demands for the reliable and automatic detection and classification of pollen, which enabled a comprehensive dataset of ca. 22,000 individual pollen grains from 18 families, 36 genera, and 37 species [33]. An interesting on-side study was performed by Bohlmann et al. in a rural forest site in Finland by investigating the optical properties of pollen in the atmosphere using a multiwavelength Raman polarization lidar (the light detection and ranging method). They presented the potential of the particle depolarization ratio to track pollen grains in the atmosphere [34]. In a following study from the same group, the authors used the same method with a novel algorithm to characterize and classify pure pollen, and thereby estimated their optical properties [35]. A comprehensive insight into the biochemistry of pollen was reported by Kendel et al. using Fourier Transform (FT-)Raman and FT-IR spectroscopy for the analysis of different pollen species [36], thus demonstrating the potential of these complementary techniques. While FT-Raman spectroscopy provided mainly information on the strong Raman scatterers sporopollenin and carotenoids in the grain walls, FT-IR spectra tend to be more sensitive to the components of the grain interior, such as lipids and carbohydrates. In an even larger-scaled study, Bağcioğlu et al. used seven different IR and Raman methodologies to highlight different chemical aspects of 15 conifer pollens [37]. They discussed the differences between the species in sporopollenin as the major constituent of the grain walls, as well as the presence of lipids and proteins in the corpus of the conifer pollen grains. The study did not include imaging approaches, and only measured the Raman spectra at two defined positions on a pollen grain. As the authors state, the layered structure leads to the chemical heterogeneity of the pollen grains; hence, the spectra differ when the depth of the laser focus is changed. Almost all Raman or IR studies lack imaging approaches, and are therefore confined to single spectrum representing an average spectrum or even only a small area of a pollen grain. The local molecular variations are especially prominent in Raman spectra recorded on confocal microscope setups with a high NA objective [31,37]. While IR technologies typically lack sufficient spatial resolution to differentiate different pollen layers, and hence are based on average spectra [17,38], Raman imaging can provide detailed molecular maps of a pollen grain with valuable information on sub-grain resolution [24].

In this study, pollen grains of different birch species, a typical tree species native to Germany, were subjected to Raman spectroscopic imaging. Birches are frequently found in mixed forests, tree alleys, parks, and open spaces at field margins, and are therefore ubiquitous. The monitoring of changes in the grains of different birch species over the years with changing climatic conditions (e.g., changes in the mean annual temperature or the amount of precipitation) can be important, and important for the consideration of the allergenic potential. Therefore, the aim of this study is the evaluation of the Raman spectroscopic imaging of single birch pollen grains, with the main focus being the investigation of their layered structure by imaging and analyzing their spectral Raman signatures. The results of seven different birch pollen species collected at two different locations in Germany are presented. The hierarchical cluster analysis (HCA) of the Raman images visualizes the grain wall in several layers, as well as the inner core. Besides sporopollenin as the main constituent of the outer layer (exine), carbohydrate bands reveal the presence of starch in the inner vegetative cell. A multiple-curve resolution algorithm further revealed differences in the concentration of sporopollenin, carbohydrate, lipid and protein components in the pollen species.

## 2. Results and Discussion

**Figure 1:**
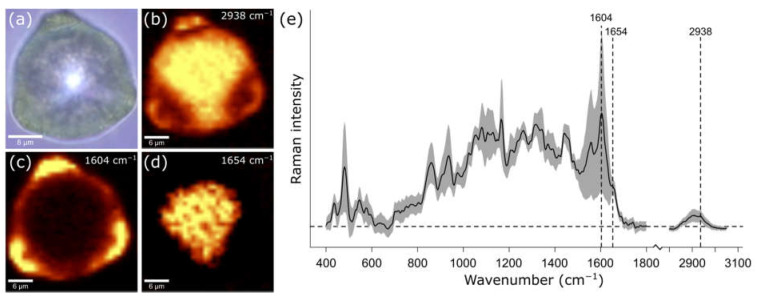
Imaging data of a *Betula ermanii* pollen grain in (**a**) a bright-field image, (**b**) a Raman intensity map over the CH stretching band between 2800 to 3010 cm^−1^, (**c**) a Raman intensity map over 1570 to 1620 cm^−1^ representing sporopollenin in the grain wall, (**d**) a Raman intensity map over 1620–1680 cm^−1^ as a typical protein and lipid band. The brighter the color in the intensity maps, the higher the intensity of the respective spectral band. (**e**) The mean spectrum in black and standard deviation in grey of the entire Raman image.

The grains of seven different birch species sampled in the Forest Botanical Garden and Arboretum of the University of Göttingen and the Botanic Garden Jena of the Friedrich Schiller University Jena were subjected to Raman imaging. Figure 1a shows a brightfield image of a *Betula (B.) ermanii* pollen grain, with the typical triangular-like shape of birch pollen due to three visible protrusions. Intensity-based Raman maps at three distinct Raman bands for the same pollen reveal a more detailed structure of the grain than is simply observed in the brightfield image. Figure 1b displays the Raman intensity map of the CH stretching region between 2800 and 3010 cm^−1^, which is present in nearly all biological compounds. A higher brightness in the Raman map indicates a higher Raman signal intensity of the band at the specific location. For example, while the core of the pollen grain appears bright, indicating a higher concentration of CH, the three protrusions have a reduced intensity, indicating that the CH concentration is lower in comparison to the core. On the other hand, the protrusions are prominently visible in Figure 1c, in which the sample distribution of the 1570 to 1620 cm^−1^ band region is shown. This spectral band indicates the presence of sporopollenin with its strongest band at 1604 cm^−1^, and thus marks the exine of the pollen grain. Contrarily, Figure 1d highlights the inside of the pollen grain by mapping the distribution of the spectral region between 1620 to 1680 cm^−1^, where typically the amid I band of proteins and the C=C stretch vibration of lipids around 1654 cm^−1^ is present. Figure 1e represents the mean spectrum with its standard deviation of the entire recorded Raman image of the pollen grain with previously assigned band positions.

**Figure 2:**
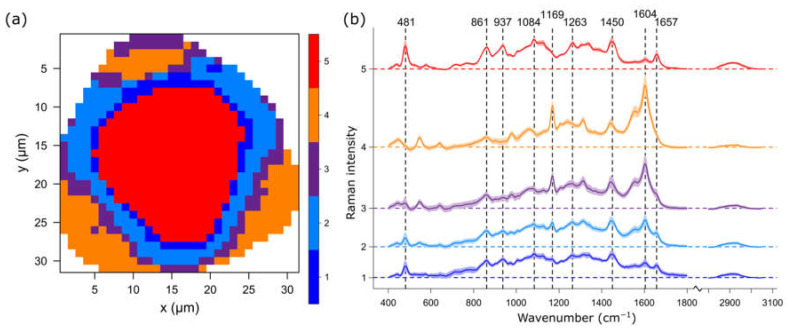
Representative cluster analysis of a *Betula ermanii* pollen grain. (**a**) HCA cluster image and (**b**) cluster spectra with the same color coding. HCA clustering was performed on, in total, 40 grains from seven different birch species. Five clusters described the pollen grain in detail, while clusters 1–4 represent four layers surrounding the inner core (cluster 5). The mean spectrum of cluster 4 reveals a high content of sporopollenin in the outside layer, which decreases in layers closer to the inner core (cluster 1–3). The mean spectra of cluster 5 are dominated by carbohydrate bands.

It is apparent that the fingerprint region between 400 and 1800 cm^−1^ includes a more comprehensive band profile than that which is presented in the intensity maps. A better insight can be gained by analyzing Raman images with spectral clustering algorithms, in which spectra are grouped based on a particular metric, e.g., Euclidian distance or Manhattan distance, of similar band profiles and averaged into cluster mean spectra, while the spatial distribution based on the chemical information is revealed in a color-coded cluster image (Figure 2). Five clusters are characterize the pollen grain in sufficient detail, in which four layers surrounding the inner core are revealed. The core of the pollen grain is represented by the red cluster 5. The corresponding cluster spectrum is dominated by carbohydrate bands originating from starch at 481, 861, 937, 1084 and 1263 cm^−1^ [28]. Starch, with amylopectin and amylose as its two naturally occurring components, is known to form granules in the vegetative cell of pollen [25]. Further bands, 1450 and 1657 cm^−1^, can be assigned to CH_2_/CH_3_ deformation, as well as amid I and C=C stretch vibrations, which are commonly representative of proteins and lipids. The orange cluster 4 is highly different, with the most intense bands at 1604 and 1169 cm^−1^, which can be associated with sporopollenin as the building block of the exine. The cluster is located at the three protrusions seen in the brightfield image (Figure 1a). Clusters 1 to 3 seem to be mixtures of clusters 4 and 5 in varying ratios. The purple cluster 3 shows a still-high content of sporopollenin, and can be found on the outside of the grain, most likely representing the exine in combination with the orange cluster. The light-blue cluster 2 already has more influence from proteins and lipids, as the band at 1650 cm^−1^ is more pronounced than that in cluster 3. Even more pronounced bands at 1650 cm^−1^ and at 1004 cm^−1^, typical for phenylalanine ring breathing, are visible in the dark-blue cluster 1, where the carbohydrate vibrations are also stronger. Both light- and dark-blue clusters form layers surrounding the red inner core. Therefore, it is reasonable to assume that they represent the intine. Roughly summarized, clusters 1 and 2 relate to the intine, cluster 3 and 4 to the exine, and cluster 5 relates to the vegetative cell. However, the clear separation of the exine, intine and interior is not straightforward, as the spectral profiles overlap due to their similar chemical structures, e.g., of carbohydrates. For example, the intine is typically composed of cellulose and pectin, which are—akin to starch—polysaccharides, and are composed of monosaccharides. Due to their having the same functional groups, the differences in the band profiles are only minor wavenumber shifts. Because cellulose bands cannot be identified in the recorded spectra, they might be strongly overlapping with the abundant starch bands in the vegetative cell, making it potentially challenging to correctly differentiate the intine [39]. Furthermore, although the measurements were preformed confocally, information of components related to sporopollenin might still be detected in cluster 1 and 2 of the intine due to a high scattering cross section of the compounds. Of note, three clusters also provide enough separation to distinguish the exine, intine and the interior; however, the authors think that five clusters provide a better and more detailed representation (see Supporting Information Figure S1 for three clusters).

**Figure 3:**
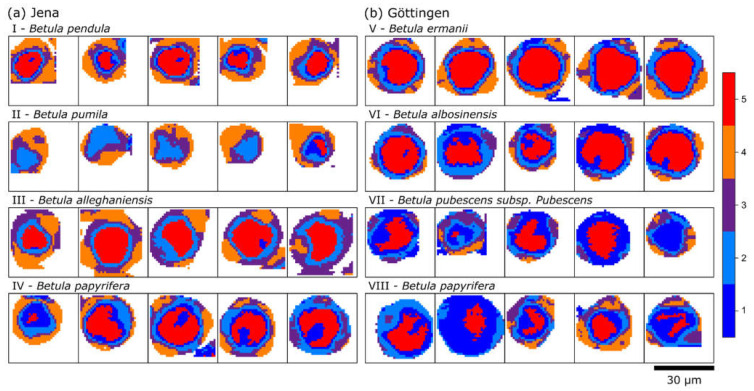
HCA cluster images of 40 pollen grains from two collection sites at (**a**) the Botanic Garden Jena of the Friedrich Schiller University Jena, and (**b**) the Forest Botanical Garden and Arboretum of the University of Göttingen. Five pollen samples from four different *Betula* species were recorded for each site. The five clusters describe the outer layers and inner core of each pollen grain, and correlate to the cluster spectra in Figure 2b.

Summarizing the spectral data, birch pollen grains have high sporopollenin and carbohydrate contents and low amounts of components related to proteins and lipids. There are no bands of carotenoids visible, which even in a low amount would produce a clear signal due to resonance enhancement. This observation is consistent with literature, in which birch pollen—as an anemophilous species—is known to have high carbohydrate but low carotenoid and protein contents [36]. The mean spectra in Figure 2b are based on the HCA clustering of, in total, 40 pollen grains from two different collection sites and seven different birch species of the genus *Betula* (B.). The corresponding cluster maps are presented in Figure 3.

Each tile represents one of five measured pollen grains for each species, whose names are displayed on top. As the tiles have the same dimensions, grains from *B. pendula* and *pumila* (a (I, II)) are the smallest, with a diameter of ca. 20 µm. *B. pubescens* (b (IV)) is around 25 µm, and the other grains are between 30 and 35 µm in diameter.

Analyzing the occurrences and distributions of the clusters, a clear difference in the orange cluster 5 and purple cluster 3, both representative for a high sporopollenin concentration, can be seen. Pollen grains from a (I–IV) and b (V) have clearly defined orange clusters on the outside of the grain, although in varying amounts. In most cases a purple-clustered layer separates the orange from the inner clusters of the grain. In contrast, in grains from b (VI, VII and VIII), less sporopollenin (orange cluster) can be seen. Here, the purple clusters do not form a layer but group on the outside in the protrusion of the grains, as the orange cluster does in the other pollen. This might indicate that the wall of these three birch pollen grains of b (VI, VII, VIII) have a reduced stability, as sporopollenin is an extremely resilient structure against physical and chemical stressors, especially against solar UV radiation [37]. Furthermore, in these three grains, the dark-blue cluster is more pronounced than in the other grains, in which more carbohydrate contributions are present in the corresponding mean spectrum, which surrounds the red cluster of the vegetative cell. The aforementioned difficulty of assigning certain clusters to specific layers is now even more obvious. While the exine of pollen grains a (I–IV) and b (V) can be assigned to the orange and purple cluster, the exine in the grains b (VI, VII and VIII) seem to be represented by the purple and light-blue clusters. It is noteworthy that as the pollen grains were measured dried, the folding of the pollen wall occurs. Based on an effect termed harmomegathy, the apertures and pores of pollen are closed off in order to minimize desiccation [40]. This could lead to a distorted view of the layered structure of pollen grains.

However, in Figure 3a, higher sporopollenin contents were observed in samples collected in the Botanic Garden Jena of the Friedrich Schiller University Jena compared to those of the Forest Botanical Garden and Arboretum of the University of Göttingen. Given the limited number of samples, it is not possible to verify whether this difference is indeed due to the different environmental conditions in these two locations. Nevertheless the difference in sporopollenin content can be seen when comparing pollen grains from Figure 3a (IV) and Figure 3b (VIII), which are from the same species of *B. papyrifera,* but were collected at the two different locations.

**Figure 4:**
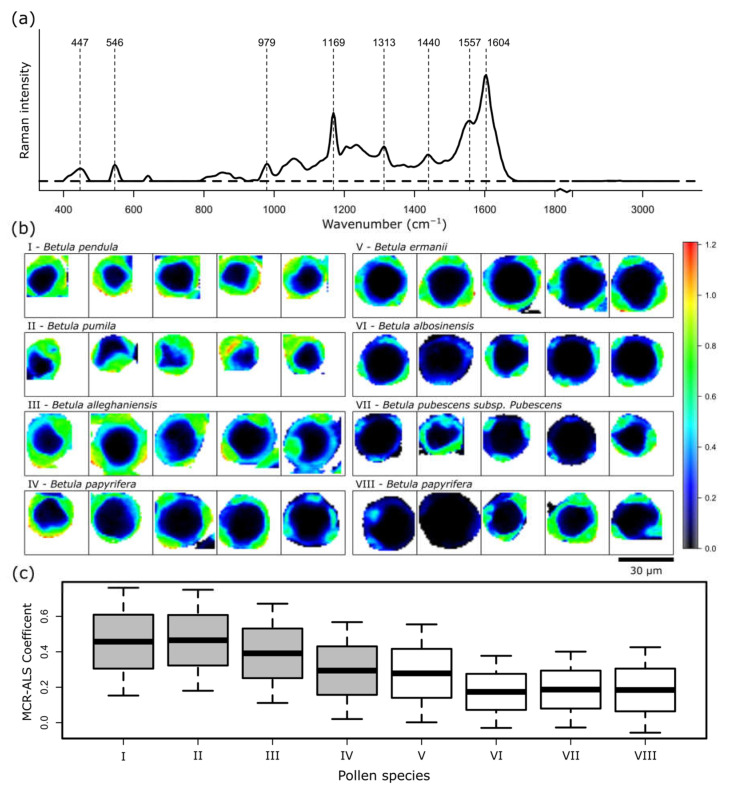
MCR-ALS results for the component representing sporopollenin. (**a**) Calculated spectrum, (**b**) spatial distribution and (**c**) mean concentration of the components of seven different pollen species. Pollens (**I**–**IV**) were collected in the Botanic Garden Jena of the Friedrich Schiller University Jena, and pollens (**V**–**VIII**) were collected in the Forest Botanical Garden and Arboretum of the University of Göttingen.

In order to gain quantitative metrics for the identification of components in the pollen grains, a multiple-curve resolution-alternating least squares (MCR-ALS) algorithm was used [41,42]. Here, input spectra and concentrations are fitted iteratively to the dataset in order to calculate the underlying pure component spectra and corresponding concentrations as a non-negative linear combination. Five spectra were identified to sufficiently describe the dataset, and are presented in the Supplementary Information Figure S2. Three components are presented in more detail in the following analysis. Figure 4 shows the results of a spectrum, which can be assigned to sporopollenin. Nearly all of the bands in the calculated spectrum in Figure 4a can be correlated to vibrations of phenylpropanoic acids, such as ferulic acid, *p*-coumeric acid or sinapic acid (1604, 1557, 1169, 979, 447 cm^−1^), which are the building blocks of sporopollenin [36,37]. However, the structural composition of sporopollenin is still not completely understood. The band at 546 cm^−1^ reveals acyl group vibrations and 1440 cm^−1^ of CH_2_/CH_3_ deformation vibrations. Figure 4b demonstrates the concentration of the component in the imaging domain. Again, the grain wall features the highest concentration, while in the inside of the grain the sporopollenin component is not detected. In order to visualize the quantitative results, the concentration of each grain was averaged and plotted in a box plot, in Figure 4c. The graph supports the previous assumption that pollen grains from the Forest Botanical Garden and Arboretum of the University of Göttingen have a lower sporopollenin content than grains from the Botanic Garden Jena of the Friedrich Schiller University Jena. Only *B. ermanii* from the Forest Botanical Garden and Arboretum of the University of Göttingen show an elevated sporopollenin content. In accordance, when comparing the two batches of *B. papyrifera*, a slight increase in sporopollenin can be seen in the samples collected in the Botanic Garden Jena of the Friedrich Schiller University Jena. The production of phenylpropanoic acids in plants has been shown to depend on environmental conditions, and can be correlated to e.g., the UV-B exposure of the parent plant [36].

**Figure 5:**
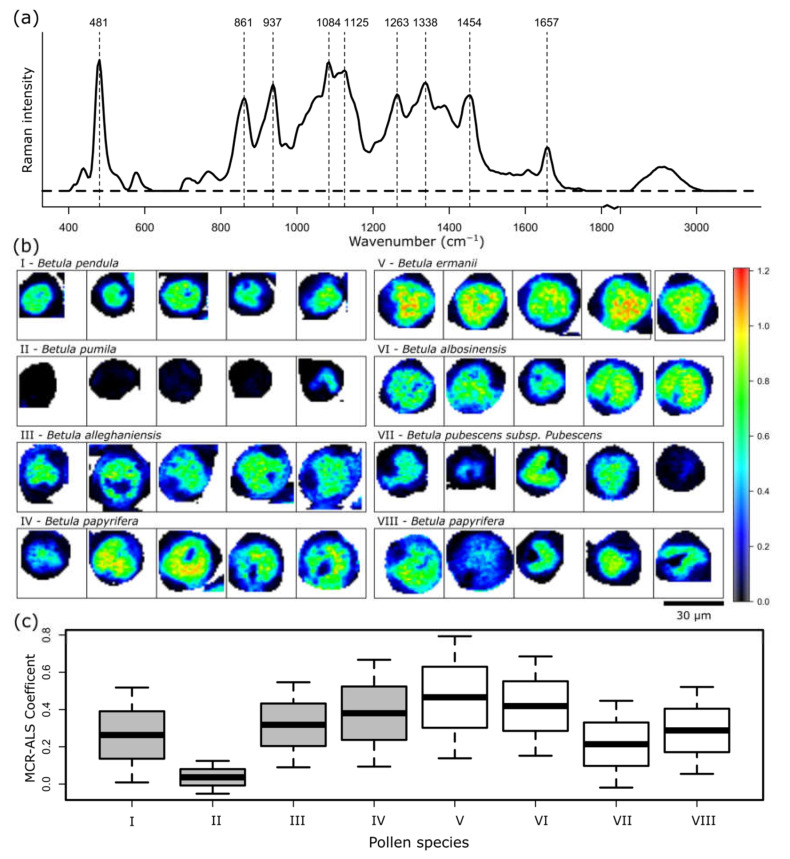
MCR-ALS results for the component representing carbohydrates. (**a**) Calculated spectrum, (**b**) spatial distribution, and (**c**) mean concentration of the components of seven different pollen species. Pollens (**I**–**IV**) were collected in the Botanic Garden Jena of the Friedrich Schiller University Jena, and pollens (**V**–**VIII**) were collected in the Forest Botanical Garden and Arboretum of the University of Göttingen.

Another component found by the MCR-ALS algorithm can be assigned to carbohydrates (Figure 5). The bands at 481, 861, 937, 1084, 1263 cm^−1^, as stated above, as well as the bands at 1125 and 1338 cm^−1^ can most likely be assigned to the starch components amylopectin and amylose. Compared to the red cluster spectra in Figure 2b, the bands of carbohydrates seem to be more clearly pronounced, as shown in Figure 5a. Interestingly, the band at 1454 cm^−1^ is shifted to higher wavenumbers. This, as well, could indicate the presence of starch, as the CH_2_ twisting and CH bending vibrations of starch occur at 1462 cm^−1^, while the CH_2_/CH_3_ deformation vibrations in lipids and proteins are typically at lower wavenumbers around 1445 cm^−1^ [28].

As expected, the carbohydrates are highly dense in the inner part of the pollen grain, as seen in Figure 5b, and indirectly reveal the location of the vegetative cell. Apparently, *B. pumila* (II) has only a really low amount of carbohydrates, which is also shown in Figure 5c in the averaged MCR-ALS coefficient value of 0.04. The concentration of carbohydrates does not correlate with the collection site, and varies more between the species than is seen in the sporopollenin content. The MCR-ALS coefficients for the *B. papyrifera* from the Botanic Garden Jena of the Friedrich Schiller University Jena are, at 0.38, higher than those from the Forest Botanical Garden and Arboretum of the University of Göttingen, with 0.29. However, the dataset is too small to give a conclusion about species-dependent correlations.

**Figure 6:**
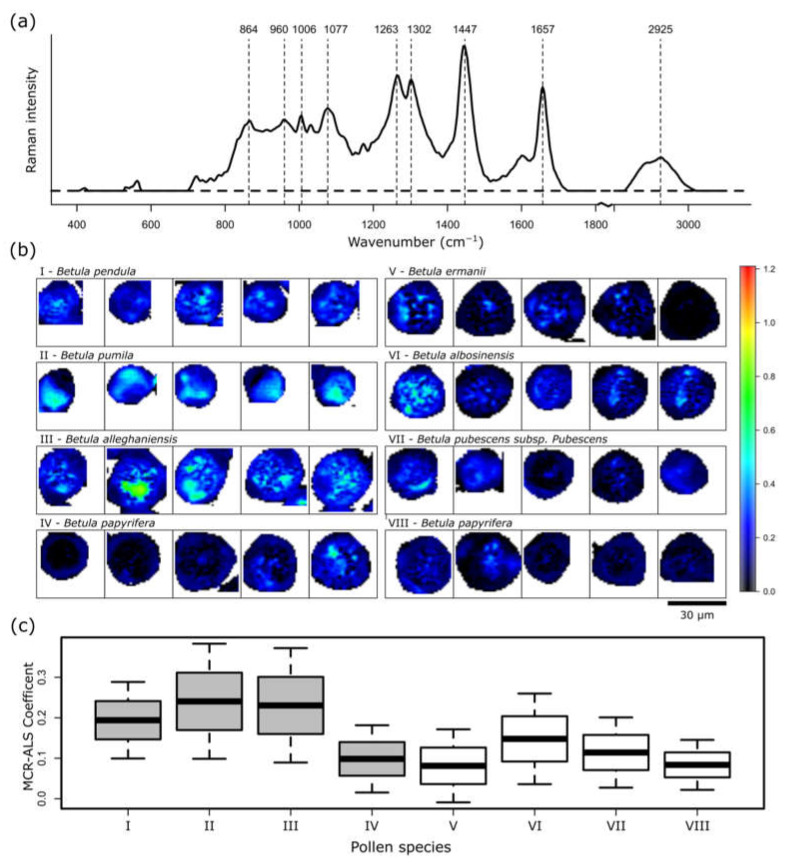
MCR-ALS results for a component representing lipid and protein content. (**a**) Calculated spectrum, (**b**) spatial distribution and (**c**) mean concentration of the components of seven different pollen species. Pollens (**I**–**IV**) were collected in the Botanic Garden Jena of the Friedrich Schiller University Jena, and pollens (**V**–**VIII**) were collected in the Forest Botanical Garden and Arboretum of the University of Göttingen.

A new component, which was not revealed by the HCA cluster analysis, is presented in Figure 6. The spectrum is rich in lipid bands. More specifically, several bands can be assigned to phosphate group vibrations of phospholipids at 864, 960, and 1077 cm^−1^. Furthermore, 1302 cm^−1^ is characteristic for lipids. However, influences of proteins can also be seen due to the presence of the phenylalanine ring breathing band at 1006 cm^−1^, and the maximum at 2925 cm^−1^ in the CH stretching region also indicates protein contributions. Some bands can be related to both lipids and proteins. 1263 cm^−1^ can indicate fatty acids, and the amid III band of proteins and 1657 cm^−1^ C=C double-bond vibration of lipids and the amid I band of proteins. Again, the band at 1447 cm^−1^ presents CH_2_/CH_3_ deformation vibrations.

In all of the samples, the representation in the imaging domain reveals that this lipid- and protein-rich component is located in the inner layers of the grains in a rather discrete form, especially in pollen grains of *B. alleghenensis* (b III). Furthermore, *B. pumila* (b II), which seems to exhibit nearly no carbohydrates, has a higher occurrence of this component. The presence or absence of the lipid-rich component is discussed as an indicator of the germination state of the pollen grain [43]. A high phospholipid content might indicate the accumulation of this membrane component, which is necessary for the growth of the pollen tube. Furthermore, it might reveal the position of the generative cell, which later in the process of germination separates into the sperm cells. The averaged representation in Figure 6c indicates that the pollen grains from the Botanic Garden Jena of the Friedrich Schiller University Jena have a higher lipid content than the grains from the Forest Botanical Garden and Arboretum of the University of Göttingen. Only *B. papyrifera* from Jena (IV) has a low amount, which correlates with *B. papyrifera* from Göttingen (VIII).

## 3. Material and Methods

### 3.1. Sample Collection

The grains of 10 single trees of seven different birch species (*Betula (B.) pumila*, *B. alleghaniensis*, *B. papyrifera*, *B. ermanii*, *B. albosinensis*, *B. pubescens*, and *B. pendula*) were sampled at flowering time (April 2019) in the Forest Botanical Garden and Arboretum of the University of Göttingen, Germany, and the Botanic Garden Jena of the Friedrich Schiller University Jena, Germany, in spring 2019. A botanist confirmed the species. The collected birch grains were stored immediately after collection at −20 °C until purification.

### 3.2. Sample Preparation for the Raman Analysis

The frozen grains were mortared using a pestle. Two milliliters of distilled water were added to the mortared pollen of each frozen birch catkin, and were suspended. Sieving (2 µm) was used to remove large non-specific components of the grain pollen suspension, and then the suspension was centrifuged at 14,000 rpm at 4 °C. The deposed pellet was stored at −20 °C until analysis for further processes. Using a microscope (400-fold), the purity of the pellet was verified (>95%). For the Raman measurement, the pollen grains were dried under ambient conditions on a CaF_2_ slide. This was necessary due to the hydrophobic property of the exine, which is commonly due to the presence of pollenkitt.

### 3.3. Raman Spectroscopic Measurements

The Raman measurements were performed on a confocal Raman microscope alpha300 R (WITec, Ulm, Germany) equipped with a 785 nm narrow-band diode laser (Toptica Photonics, Gräfelingen, Germany). The spectrometer, equipped a 300 g/mm grating, allows us to cover the wavenumber region between 100 to 3300 cm^−1^ at a spectral resolution of approx. 6 cm^−1^. Raman spectra were recorded with an integration time of 0.5 s using a 50× Zeiss objective with 0.9 NA. The prepared pollen grains were placed on a CaF_2_ slide, and the Raman images were recorded on individual grains with a step size of 1 µm.

The data analysis of the recorded Raman images was performed in R with *hyperSpec* as a main package [44,45]. Several preprocessing steps were performed, including wavenumber calibration, with paracetamol spectra as references, and interpolation. The rubberband method was used to correct the background, and afterwards a cosmic spike correction algorithm [46] was used. An area normalization over the range of 400 to 3100 cm^−1^ was implemented. In order to reduce the dataset, spectra not exhibiting information from the pollen grains were removed, e.g., of the surrounding CaF_2_ slide, and a correlation function with a threshold of 0.75 was implemented as a quality control to remove outliers. A Savitzky–Golay function from package *parcma* was chosen to smooth the spectra with a quadratic filter order and filter length of 9 [47].

An HCA based on a Pearson distance calculation and the Ward clustering algorithm of the entire dataset grouped the spectra into clusters of the same spectral profile. In a false-color representation, the calculated weighted mean spectra of the clusters can be presented in the imaging domain, revealing spectral differences within one pollen grain. Additionally, an MCR-ALS algorithm was chosen to calculate the pure components and their abundances in a quantitative approach [41]. In an alternating fashion, pure spectra and concentration profiles were optimized until a convergence criterion was achieved [42]. The five cluster mean spectra from the HCA were used as input starting spectra, and non-negativity constraints for both the spectra and the concentrations were set.

## 4. Conclusions

With this study, we were able to establish a method to characterize the components of pollen grains using Raman spectroscopic imaging. The different layers of pollen grains were identified and visualized based on their biochemical signatures. The established method allows the detailed analysis of single grains, which provides novel information for future studies about, e.g., differences in growth or germination processes. Raman spectroscopy can provide spatially resolved, label-free information non-destructively and rapidly, enabling the analysis of very rare palynological specimens. Moreover, through the development of more automated and compact systems, grains could also be sampled in field studies and tagged directly to individual plants for a more precise investigation of ecological conditions. Furthermore, general investigations are necessary, especially against the background of the changing climatic conditions. We must clarify how climate elements and factors change, and to what extent this leads to the detected differences.

## Supplementary Materials

Supplementary Information is available. The following are available online at https://www.mdpi.com/article/10.3390/ijms23095112/s1.
