# Supplementary material for: Revealing the Chemical Composition of Birch Pollen Grains by Raman Spectroscopic Imaging
Source: Int J Mol Sci. 2022 May 4;23(9):5112. doi: 10.3390/ijms23095112 (PMC9101400; doi:10.3390/ijms23095112)
Supplement: Supplementary file 1 [file ijms-23-05112-s001.zip › ijms-1685458-supplementary.pdf]

Revealing the chemical composition of birch pollen grains by Raman spectroscopic imaging

- Supplementary Information –

Clara Stiebing[1], Nele Post[2], Claudia Schindler[3], Bianca Göhrig[3], Harald Lux[3,4], Jürgen Popp[1,5], Astrid Heutelbeck[3], and Iwan W. Schie[1,2,*]

[1]Leibniz Institute of Photonic Technology (Leibniz-IPHT), Albert-Einstein-Straße 9, 07745 Jena, Germany
[2]Department of Medical Engineering and Biotechnology, University of Applied Sciences Jena, Carl-Zeiss-Promenade 2, 07745 Jena, Germany
[3]Institute of Occupational, Social and Environmental Medicine, Jena University Hospital, Erlanger Allee 103, 07747 Jena, Germany
[4]Department of Psychiatry, Psychotherapy and Psychosomatic Medicine, Brandenburg Medical School, 16816 Neuruppin, Germany
[5]Institute of Physical Chemistry and Abbe Center of Photonics, Friedrich Schiller University Jena, Helmholtzweg 4,07743 Jena, Germany

*corresponding author:
Prof. Iwan W. Schie, iwan.schie@leibniz-ipht.de

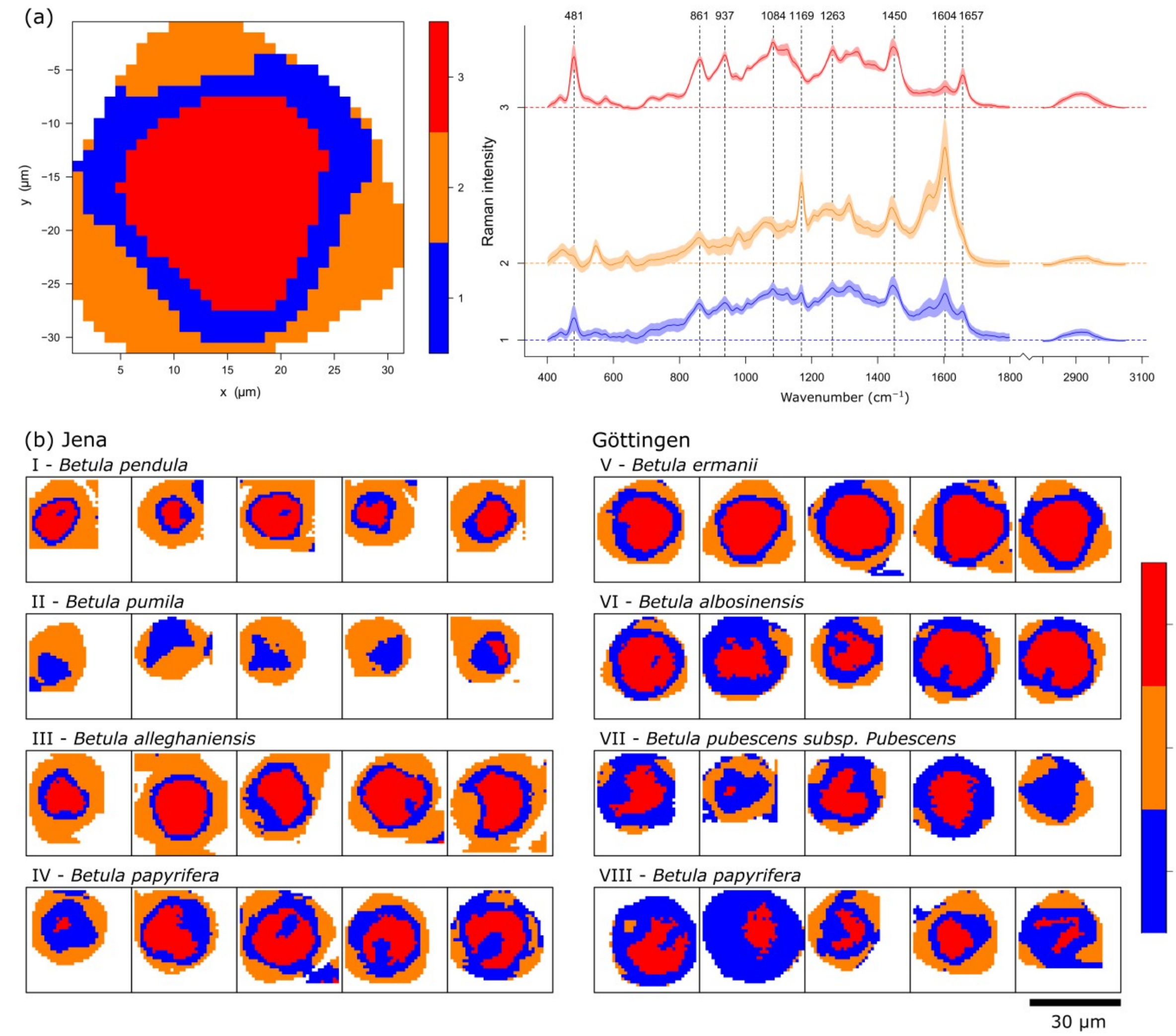

Figure S1 HCA clustering using three clusters performed on in total 40 grains from 7 different birch species (a) Representative HCA cluster image of a *Betula ermanii* pollen grain using three clusters and corresponding cluster spectra with the same color coding. (b) HCA cluster images of 40 pollen grains from two collection sites (left) at the Botanic Garden Jena of the Friedrich Schiller University Jena and (right) the Forest Botanical Garden and Arboretum of the University of Göttingen. Five pollen from four different *Betula* species were recorded for each site.

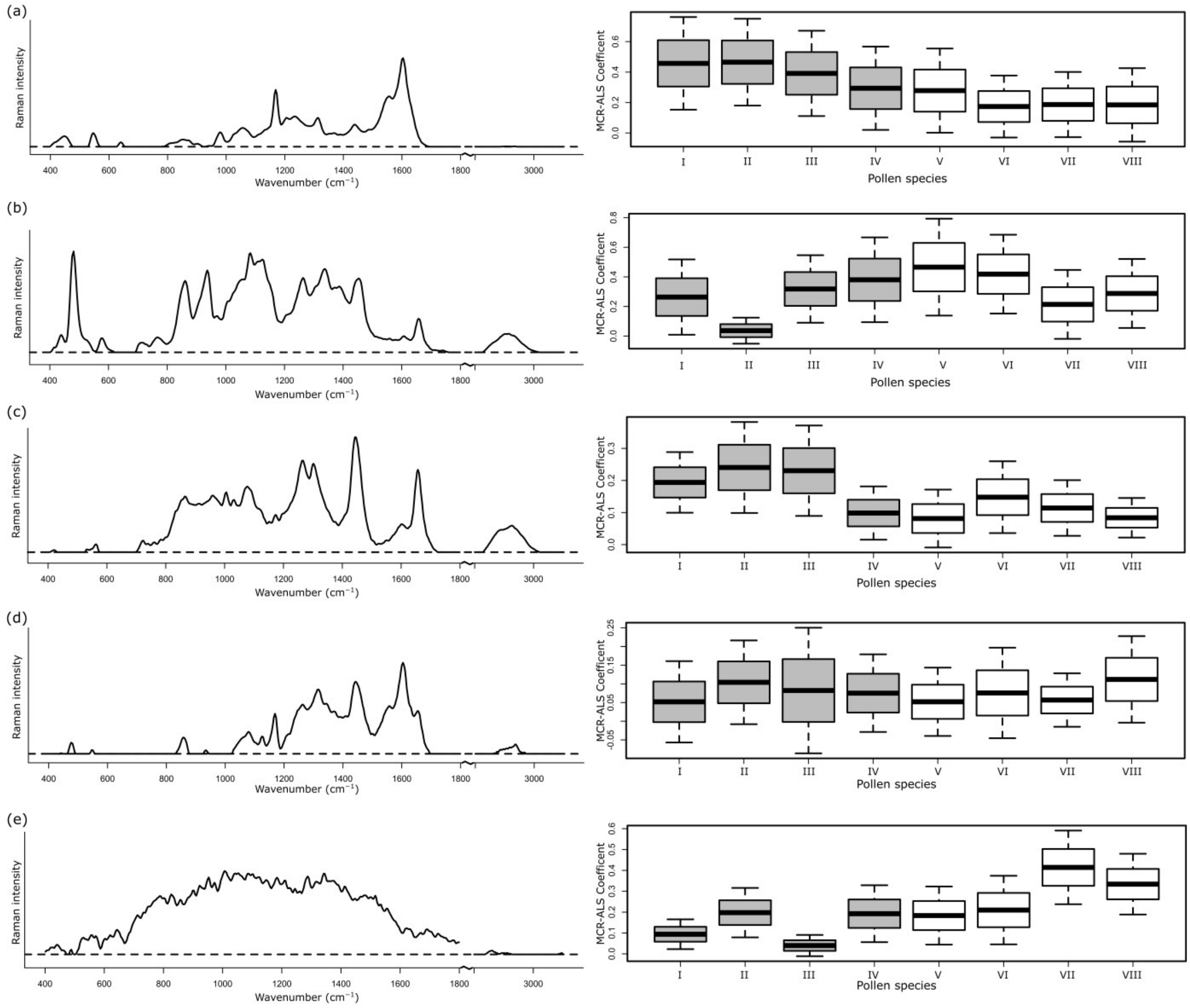

Figure S2 MCR-ALS results showing the five pure component spectra (left) and corresponding mean concentrations for each pollen species (right). Pollen I-IV were collected in Jena, V-VIII were collected in Göttingen. The components can be identified as (a) sporopollenin, (b) carbohydrates, (c) lipids and proteins, (d) mixed contribution of (a)-(c) and (e) fluorescence as a residual background.
